# Prevalence of metabolic syndrome and its components in psoriatic arthritis compared with general population, cutaneous psoriasis, and other inflammatory arthropathies: a meta-analysis

**DOI:** 10.1007/s10067-025-07637-z

**Published:** 2025-08-18

**Authors:** Sara Andreasson, Anna Södergren

**Affiliations:** 1 Department of Public Health and Clinical Medicine, Umeå University, Umeå, Sweden; 2 Department of Research and Development-Sundsvall, Umeå University, Umeå, Sweden

**Keywords:** Inflammatory arthropathies, Meta-analysis, Metabolic syndrome, Psoriatic arthritis

## Abstract

**Objectives:**

Through this meta-analysis, we aim to provide an overview and statistical synthesis of the prevalence of MetS and its components in psoriatic arthritis (PsA) compared to the general populations, patients with cutaneous psoriasis (PsO), and patients with other inflammatory arthropathies.

**Method:**

A search was conducted in Ovid Medline, Web of Science, and Scopus up to February 2024. Original articles investigating the prevalence of MetS in PsA in adults compared to one or more comparison populations were included. Bias risk was assessed by means of a funnel plot. The data was analyzed by means of a random effects model and presented in forest plots.

**Results:**

Of 1526 articles in the original search, 20 relevant were identified. Meta-analyses showed an increased prevalence of MetS in PsA compared to the general population, rheumatoid arthritis (RA), and ankylosing spondylitis (AS) (LogOR 0.93, 0.63, and 1.04, respectively). Meta-analysis showed no difference in the prevalence of MetS in PsA compared to PsO (LogOR 0.15, *I*^2^ 0.63). Meta-analysis of the prevalence of the different components of MetS in PsA compared to RA showed an increased prevalence of central obesity, hypertriglyceridemia, impaired glucose tolerance, and diabetes mellitus.

**Conclusions:**

PsA was associated with an increased risk of MetS compared to the risk in the general population, in RA and in AS, respectively. This emphasizes the importance of screening for and taking necessary measures to prevent MetS in patients with PsA.

**Table:** 

**Key Points** • *Patients with PsA have an increased risk of MetS compared to the general population as well as patients with RA or AS*. • *According to this meta-analysis, the risk of MetS is the same in patients with PsA as in patients with PsO*

**Supplementary Information:**

The online version contains supplementary material available at 10.1007/s10067-025-07637-z.

## Introduction

Psoriatic arthritis (PsA) is a chronic inflammatory joint disease with a variety of different clinical phenotypes and is commonly associated with cutaneous psoriasis (PsO). Psoriasis as a comprehensive concept has increasingly been recognized as a systemic disease with effects beyond the joints and skin. The pathogenesis of PsA is yet to be fully understood. However, the main hypothesis describes an interplay between genetic predisposition and environmental factors leading to dysfunction of immunological and inflammatory signaling pathways. For instance, obesity and hyperlipidemia are among the risk factors for the development of PsA [1, 2].

There is a well-established association between PsA and cardiovascular disease. The cardiovascular risk profile has been shown to be increased as early as at the onset of the disease [3–5], and the risk of developing cardiovascular morbidity has been found to be underestimated by conventional risk assessment tools. Moreover, previous studies indicate associations between cardiovascular risk and disease activity, choice of treatment, as well as the presence of high proinflammatory cytokines [4, 6–10].

Metabolic syndrome (MetS) defines a specific subgroup of patients at increased risk of cardiovascular disease and type 2 diabetes due to a common underlying pathophysiology of increased inflammatory activity [11]. MetS is a clustering of at least three of the conditions: abdominal obesity, insulin resistance, dyslipidemia, and hypertension, and in this syndrome the cardiovascular risk is higher than would be expected from the individual conditions alone. There are several different definitions of MetS; however, these have been found to be comparable in terms of prognosis and treatment [12]. An association between PsA and MetS has been suggested, with several studies examining the prevalence in PsA compared to other groups with somewhat diverging results [13–32].

The aim of this meta-analysis is to compile current knowledge on the prevalence of MetS and its individual conditions in PsA compared to the general population, PsO, and other inflammatory arthropathies.

## Materials and method

This systematic review is reported in accordance with the Preferred Reporting Items for Systematic Reviews and Meta-Analyses (PRISMA) guidelines. Two librarians conducted searches in the Ovid Medline, Scopus, and Web of Science databases from inception to February 2024. The search terms are presented in Appendix Figs. 1, 2 and 3. The search was limited to articles written in English or articles with an English translation. To minimize the risk of missing any article of relevance, the search terms were very wide, giving 1226 articles to screen.

The title and abstract were reviewed by two independent researchers (SA and AS) in EndNote TM × 9 software, and the articles were then either sorted into a folder for further review or discarded if they did not meet the inclusion criteria. Articles that were included for further review described in their abstracts that they were investigating the prevalence of MetS or cardiovascular morbidity in PsA. Irrelevant articles, articles lacking original data, and studies conducted in children were excluded. Articles in which the study collected its sample of patients via a dermatology clinic, resulting in a PsA population consisting of patients who all had both PsO and PsA, were excluded. Duplicates were also excluded. After this, the remaining articles were read in full and articles investigating the prevalence of MetS in patients with PsA compared to a control population were included. Studies in which the groups were matched according to any of the components of MetS were excluded.

Data from the included articles was compiled in Microsoft Excel. Data was primarily extracted regarding the sample sizes of the PsA and the control population, respectively, as well as the proportion of individuals with MetS in each group. When specified in the study, data was also extracted regarding the number of individuals with the different conditions included in MetS. Data was also extracted regarding diagnostic criteria for MetS in each study, as well as diagnostic criteria for PsA and for the conditions of the control groups when these were not general population. Finally, data was extracted regarding country of study, type of study, type of control group, sex ratio, mean age, and, if relevant, matching.

A meta-analysis was performed on the extracted data. The effect size is presented as an overall log odds ratio (logOR) and describes the probability of MetS occurring in patients with PsA compared to the respective control population (95% confidence interval (95% CI)). The same analysis was also performed on the probability of occurrence of each condition included in MetS, when sufficient data was available. *I*-square tests were used to assess heterogeneity. Chi-square tests were used to assess homogeneity. Funnel plots were used to assess publication bias. The statistical analysis was performed using IBM SPSS Statistics ver 28.0.

## Results

**Fig. 1:**
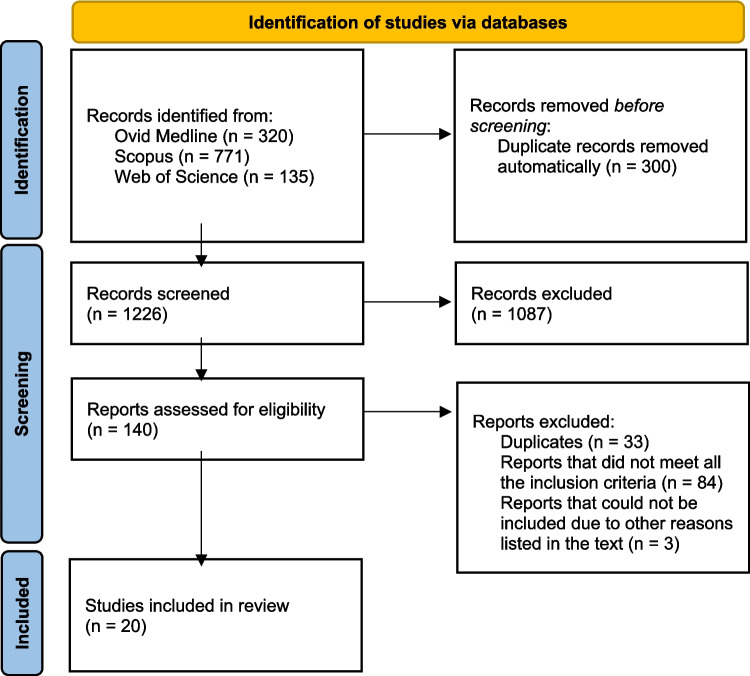
Illustration of the article selection approach

A total of 1526 publications matched the literature search. Twenty studies were included in this meta-analysis (Fig. 1) [13–32]. Three studies were excluded, despite comparing the prevalence of MetS in PsA with a control group (Fig. 1) [33–35]. Haque et al. [33] was not included as it was the only study that compared against a control group with spondyloarthritis (SpA) without PsA, and it was therefore not possible to conduct a meta-analysis on these data. Raychaudhuri et al. [34] was excluded because it used data from NAHNES III for comparison, and thus there was no information on the sample size of the control population. Tousserot et al. 2021 [35] was excluded because the control group was matched according to BMI.

A description of the 20 included studies is summarized in Supplemental Table 1. Among the included studies, the sample size ranged from 48 to 1446, with a total number of 7025 subjects. All but two studies used CASPAR criteria for the definition of PsA. The other two studies used Moll and Wright and diagnosis by a rheumatologist as definition of PsA.

### Prevalence of MetS in PsA compared to the general population

**Fig. 2:**
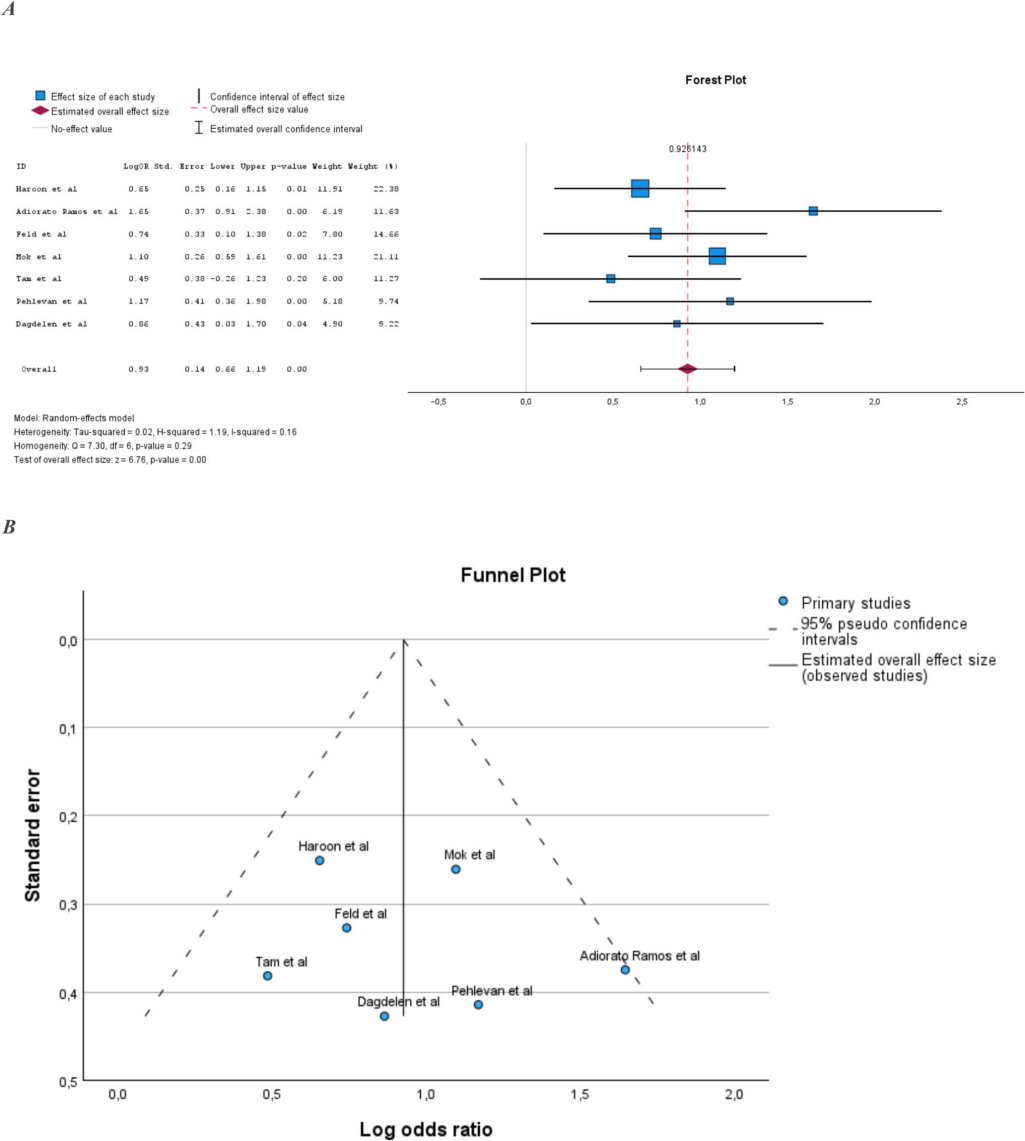
**A** Prevalence of MetS in PsA compared to the general population. **B** Illustration of publication bias in the meta-analysis comparing PsA populations and the general population

Seven studies examined the prevalence of MetS in PsA compared to the general population [13, 15–17, 20, 21, 26] (Supplemental Table 2). Sample size ranged from 100 to 383 subjects. All studies except Tam et al. [20] reported a statistically significant increase in the prevalence of MetS in PsA compared to the general population. The pooled prevalence of MetS in PsA compared to general populations was also statistically significantly increased (Fig. 2A). Heterogeneity in the meta-analysis was low and homogeneity was statistically significant (Fig. 2B).

### Prevalence of MetS in PsA compared to RA

**Fig. 3:**
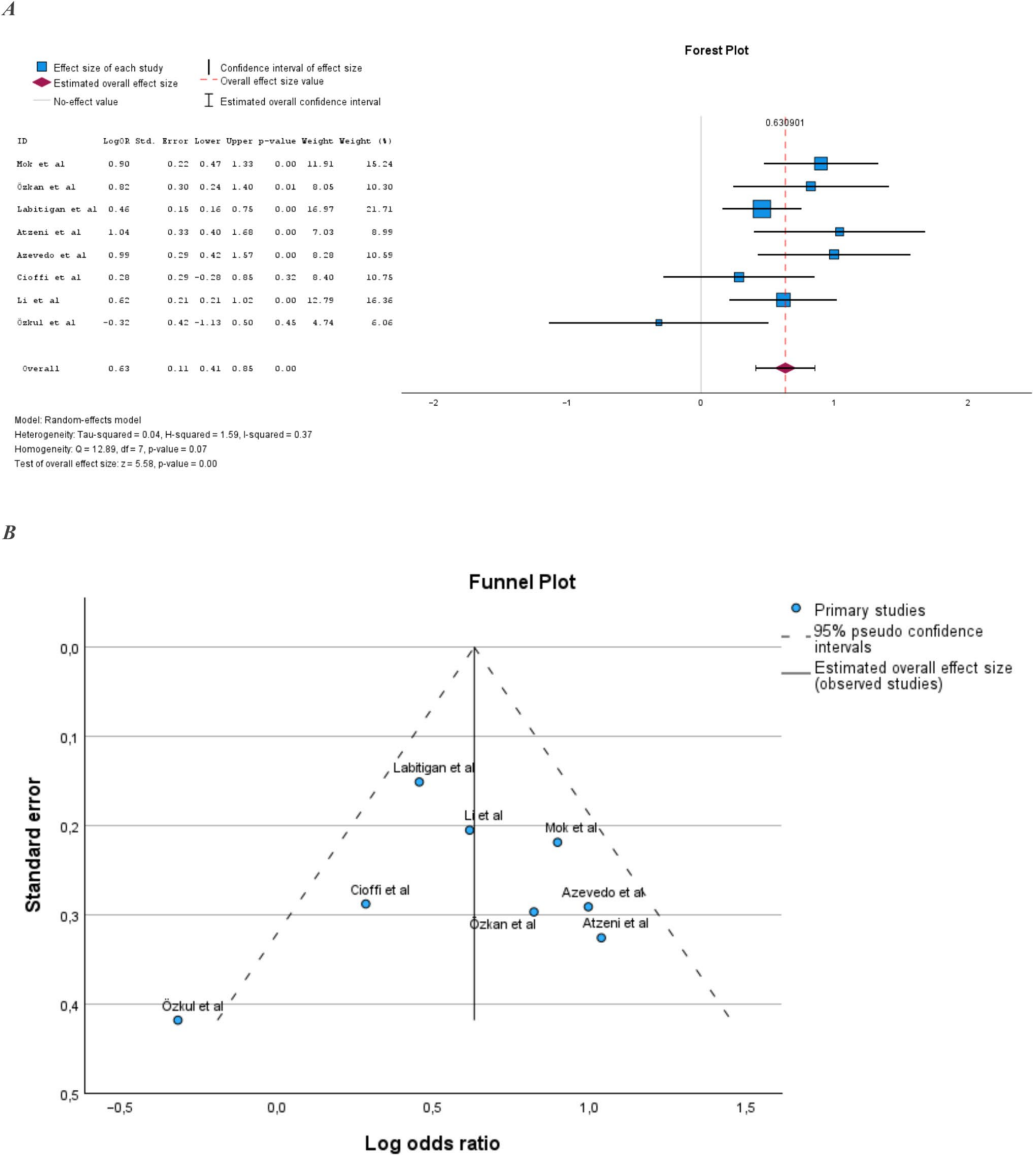
**A** Prevalence of MetS in PsA compared to RA. **B** Illustration of publication bias in the meta-analysis comparing PsA populations and RA populations

Eight studies examined the prevalence of MetS in PsA compared to rheumatoid arthritis (RA) [14, 17, 19, 22, 23, 25, 29, 32] (Supplemental Table 3). The sample size of these studies ranged from 105 to 1456 subjects. Six of the eight studies showed a statistically significant increase in the prevalence of MetS in PsA compared to RA [14, 17, 19, 22, 23, 29]. The pooled prevalence of MetS in PsA compared to RA was also statistically significantly increased (Fig. 3A). Heterogeneity in the meta-analysis was moderate and homogeneity was not statistically significant (Fig. 3B).

In [23], the prevalence of MetS differed between the PsA and RA populations by 56% and 32%, respectively. In the study’s own statistical analysis, the difference was not statistically significant (*P* = 0.059), while when included in this meta-analysis, the result was statistical significant (*P* < 0.001). In a meta-analysis where we excluded Azevedo et al. the result of the meta-analysis was still statistically significant (LogOR 0.59; 95% CI 0.36–0.82).

### Prevalence of MetS in PsA compared to AS

**Fig. 4:**
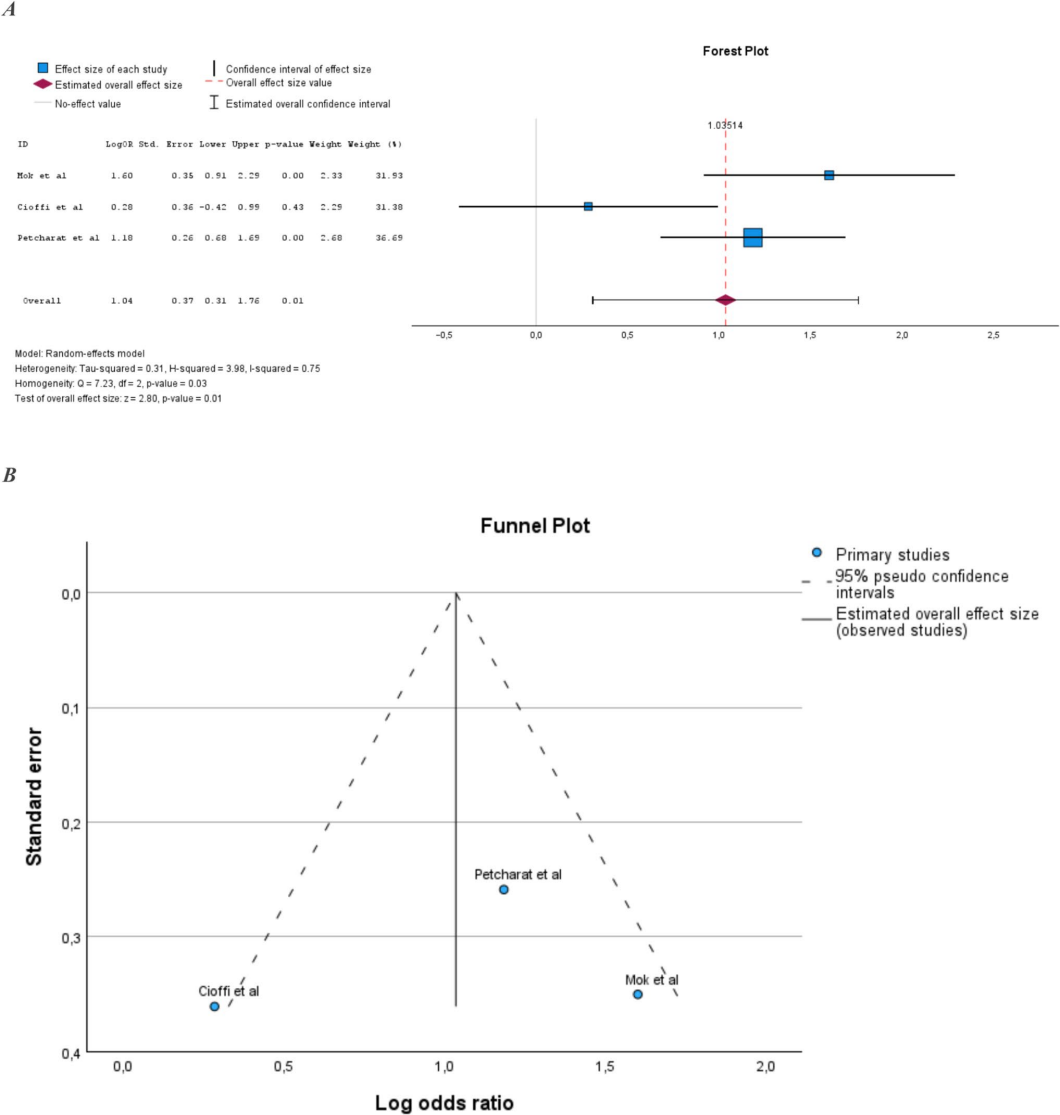
**A** Prevalence of MetS in PsA compared to AS. **B** Illustration of publication bias in the meta-analysis comparing PsA populations and AS populations

Three studies examined the prevalence of MetS in PsA compared to AS [17, 25, 31]. The studies are presented in Supplemental Table 4. Sample size ranged from 230 to 319 subjects. Two of the three studies reported a statistically significant increase in the prevalence of MetS in PsA compared to AS. The pooled prevalence of MetS in PsA compared to AS was also statistically significantly increased (Fig. 4A). Heterogeneity in the meta-analysis was high and homogeneity was statistically significant (Fig. 4B).

### Prevalence of MetS in PsA compared to PsO

**Fig. 5:**
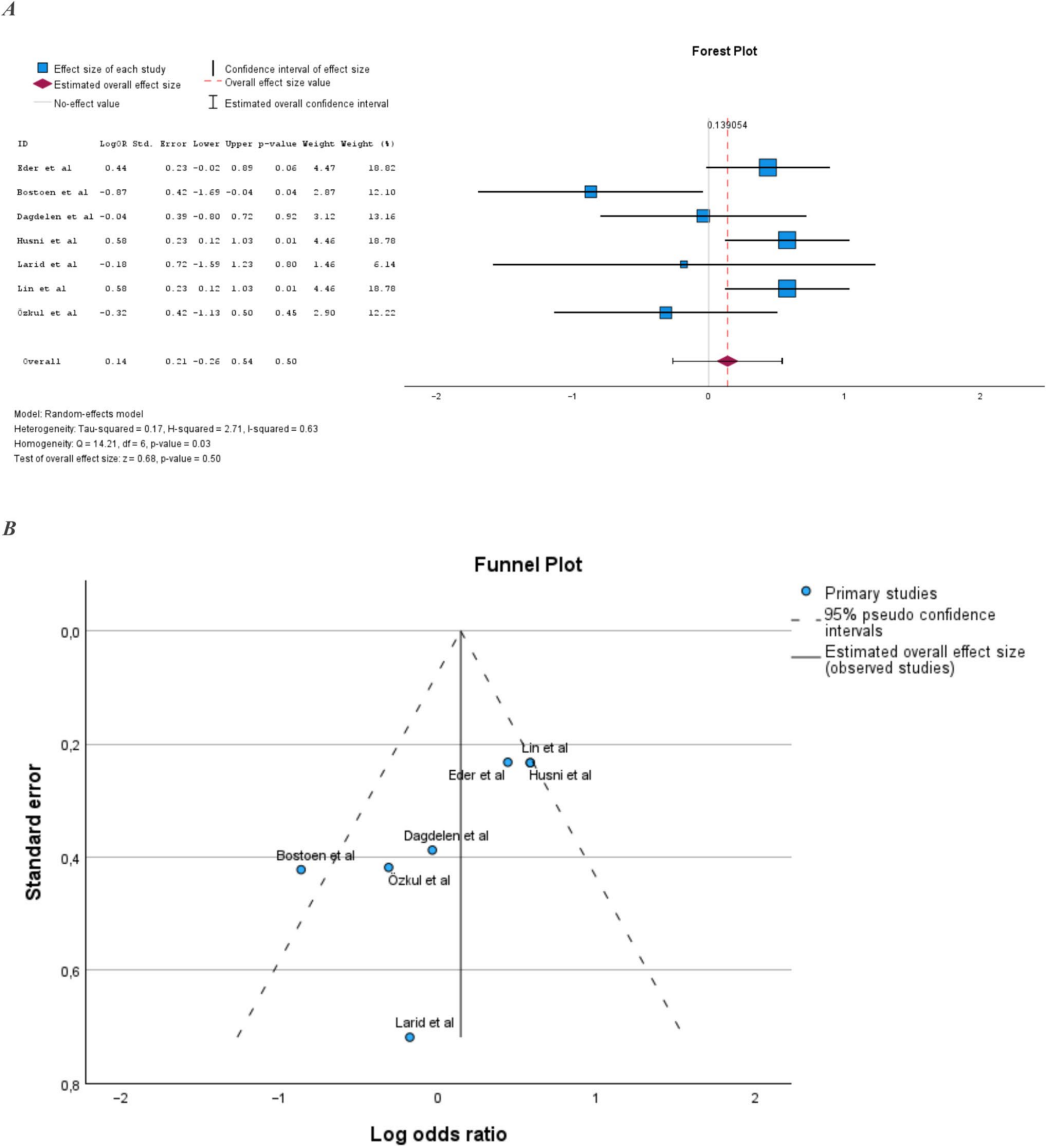
**A** Prevalence of MetS in PsA compared to PsO. **B** Illustration of publication bias in the meta-analysis comparing PsA populations and PsO populations

Seven studies examined the prevalence of MetS in PsA compared to PsO [18, 24, 26–28, 30, 32] (Supplemental Table 5). Sample size in the included studies ranged from 48 to 358 subjects. Four of the seven studies showed no statistically significant difference in the prevalence of MetS in PsA compared to PsO [18, 26, 28, 32]. Bostoen et al. [24] showed an increased prevalence of MetS in PsO compared to PsA, while Husni et al. [27] and Lin et al. [30] showed an increased prevalence of MetS in PsA compared to PsO. A meta-analysis of the pooled prevalence of MetS in PsA compared to PsO did not reveal a statistically significant difference (Fig. 5A) Heterogeneity in the meta-analysis was high but homogeneity was statistically significant (Fig. 5B).

In another meta-analysis, in which we excluded the studies that used IDF as diagnostic criterion for MetS [24, 32], a statistically significant increase in the prevalence of MetS was observed in PsA compared to PsO (LogOR 0.45; 95% CI 0.2–0.69), with low heterogeneity and without statistically significant homogeneity.

### Prevalence of the different components of MetS in PsA compared to RA

**Fig. 6:**
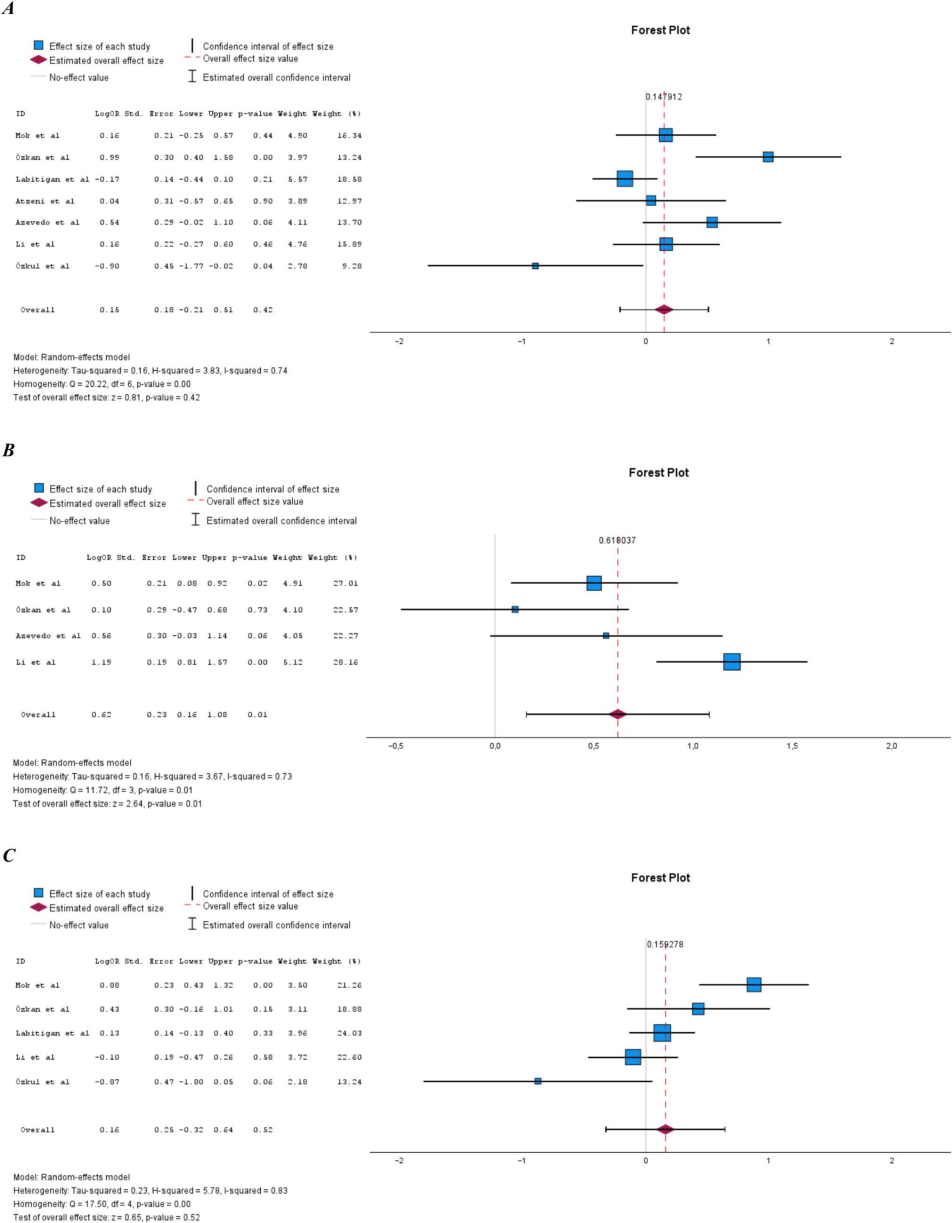

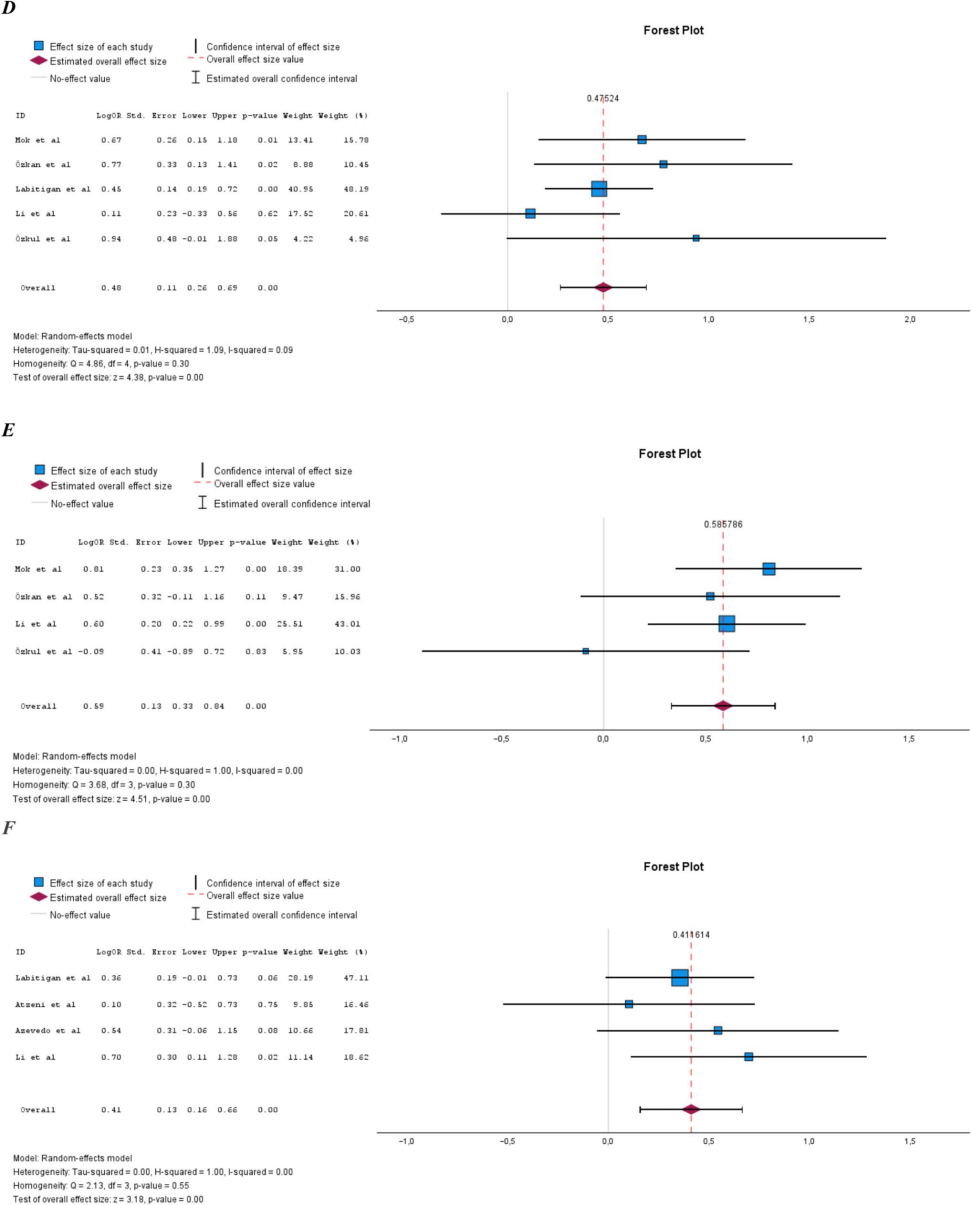
**A** Prevalence of hypertension in PsA compared to RA. **B** Prevalence of central obesity in PsA compared to RA. **C** Prevalence of low HDL in PsA compared to RA. **D** Prevalence of hypertriglyceridemia in PsA compared to RA. **E** Prevalence of impaired glucose tolerance in PsA compared to RA. **F** Prevalence of diabetes in PsA compared to RA

The overall prevalence of the different components of MetS in PsA compared to RA is shown in Fig. 6A–F. There was a statistically significant increase in the prevalence of central obesity, hypertriglyceridemia, impaired glucose tolerance, and diabetes in PsA compared to RA. The analyses comparing the prevalence of hypertriglyceridemia, impaired glucose tolerance, and diabetes had low heterogeneity and non-statistically significant homogeneity, while the reverse was true for the other components (data not shown).

## Discussion

To our knowledge, this is the first meta-analysis of studies investigating the prevalence of MetS in PsA compared to different comparison populations. Through this study, we were able to provide further support that there is an increased prevalence of MetS in PsA compared to both general populations and in other inflammatory arthropathies.

The meta-analysis comparing PsA to general populations showed a 2.5-fold increased risk of MetS in PsA, and there was no signs of publication bias or significant heterogeneity in this meta-analysis.

In the meta-analysis comparing PsA to RA, there was a 1.9-fold increased risk of MetS in PsA. However, here the analysis indicated moderate heterogeneity. Our funnel plot for assessing publication bias showed that the study by Özkul et al. [32] fell outside the cut-off, which could be explained by the small sample size of that study. Nevertheless, there was an overall symmetrical spread around the mean value in the plot. Moreover, the forest plot revealed that the study result in Özkul et al. [32] clearly diverged. In contrast to the other studies, Özkul et al. [32] showed a non-statistically significant increase in the prevalence of MetS in RA compared to PsA. There may be several reasons why the results of Özkul et al. [32] differed from the other studies. In addition to the small sample size, the sample populations differed statistically significantly in age, with the RA population being older. They also excluded patients with type 2 diabetes, which counts as insulin resistance, one of the components of MetS.

The meta-analysis comparing PsA with AS showed an increased prevalence of MetS in PsA. However, no firm conclusion can be drawn from the results of this analysis, as it only included three studies.

In the meta-analysis comparing PsA with PsO, there was no significant difference in the prevalence of MetS. There was a wide dissemination of the results of the included studies, and the analysis had high heterogeneity giving an uncertainty of the relevance of the results. The forest plot showed that there was a larger number of studies showing higher or equivalent prevalence of MetS in PsO compared to PsA; however, those studies had smaller sample sizes. This inconclusive result could be explained by the fact that the two psoriasis groups were difficult to separate clearly, as they probably share a common underlying pathophysiology. Furthermore, different definitions of MetS in the different studies could contribute to the divergent results.

The different definitions of MetS in the included studies is a potential source of bias. However, when compared, the definitions are similar or identical, except for the IDF criteria, and they have previously been found to be comparable in terms of prognosis and treatment [12]. Compared to the others, the IDF criteria give more weight to central obesity. However, in 2009, the IDF, NHLBI, AHA, and other organizations combined their criteria for MetS into the criteria known as harmonizing (IDF/NHLBI/AHA/WHF/IAS/IASO) [36]. These criteria do not give central obesity the same weight as the older IDF criteria. In the meta-analysis comparing the prevalence of MetS in PsA with PsO, it is possible to discern a correlation between the results and the criteria used to define MetS. In the cases where the IDF criteria was used, an increased prevalence of MetS was seen in PsO compared with PsA, which differs from the results of a meta-analysis by Loganathan et al. [37]. One can therefore speculate whether our result can be explained by an increased prevalence of central obesity in PsO compared to PsA. In a subgroup analysis in which we excluded the studies that used IDF criteria, an increased prevalence of MetS was instead seen in PsA compared to PsO, which is more consistent with the results in Loganathan et al. [37]. Another included study, Li et al. [29] used IDF criteria when comparing MetS in PsA and RA. Here, the results did not differ from the others.

Loganathan et al. [37] conducted a meta-analysis in 2021 that examined the prevalence of MetS in PsA compared to RA and PsO. As in our study, an increased prevalence of MetS was shown in PsA compared to RA. However, the results diverged regarding the comparison between PsA and PsO. In our study, no increased prevalence of MetS was found in PsA compared to PsO, while the opposite was shown in the meta-analysis by Loganathan et al. [37]. There may be several reasons why the results of the two meta-analyses differed. For example, the studies differed in terms of inclusion and exclusion criteria. For example, we excluded studies done on PsA populations where all included cases had concomitant PsO at baseline, possibly excluding cases with milder PsA in the present study. In addition, Loganathan et al. [37] used pooled data from all studies investigating the prevalence of MetS in PsA, which were then compared with pooled data from all studies investigating the prevalence of MetS in RA and PsO. In opposite, our study analyzed comparative data from each study in a forest and funnel plot. This approach allowed the comparison of results between each included study, which in turn gave an opportunity to more accurately evaluate statistical significancy of the meta-analysis and more clearly observe any sources of error due to different study designs. On the contrary, the study design of Loganathan et al. [37] allowed the meta-analysis to include more studies.

Indeed, it appears that there is a correlation between PsA and an increased prevalence of MetS. Moreover, this prevalence has been shown in some studies to be associated with disease activity and inflammatory activity, suggesting a common underlying pathophysiology between the two conditions [38]. For example, obesity, which is a central component of MetS as it can result in several of the other components, has previously been described as a chronic inflammatory condition with pathophysiological signaling pathways that are shared with psoriatic disease [39]. Obesity has also been shown to affect the risk of developing PsA as well as disease prognosis and treatment response [40]. In addition to this correlation, the cytokines activated in obesity and PsA have also been shown to be involved in endothelial cell dysfunction and thus the development of atherosclerotic plaques [41].

It is also of importance to mention the impact of different treatments on the occurrence of MetS in PsA. First, both cyclooxygenase inhibitors and corticosteroids per se can aggravate cardiovascular risk directly and indirectly, respectively, by inducing obesity and elevating blood glucose levels. However, previous studies have shown that both increased cardiovascular risk and obesity are increased as early as at the point of diagnosis of PsA, which is before these treatments are initiated [1, 42, 43]. Second, regarding TNF inhibitors and DMARDs, adalimumab and etanercept have been shown to improve the components of MetS more efficiently than methotrexate and may reduce the occurrence of cardiovascular events [44, 45]. This is in line with the finding that obesity is associated with lower probability of achieving and maintaining minimal disease activity [46]. The individuals of the populations in the different studies had different underlying conditions, disease durations, treatments, and disease activity, which could result in an increased heterogeneity of the results. However, this diversity could also lead to a more accurate reflection of the target population.

This common underlying pathophysiology and shared response to treatment in PsA and MetS highlight the importance of monitoring and treating both disease activity and cardiovascular morbidity. Knowledge of the correlation between PsA and MetS strengthens the notion of both conditions being different expressions of the same disease. This emphasizes that both the components of MetS as well as the PsA disease activity should be closely monitored and treated. In addition, it is easy to imagine that the treatment of one may improve the other. This is in line with the EULAR’s recommendations for the treatment of PsA which emphasizes the importance of considering comorbidities such as MetS, obesity, and cardiovascular morbidity [47]. However, clinical assessment of patients with PsA may be challenging for several reasons. For example, conventional cardiovascular risk assessment tools have been shown to underestimate the cardiovascular risk in PsA [7]. Assessment is also complicated by the fact that triglycerides and HDL, as components of MetS, may rise with decreasing disease activity due to some antirheumatic drugs [48]. The impact of disease activity, inflammation, and antirheumatic drugs on the prevalence of MetS in PsA is yet to be clarified [39].

To our knowledge, this is the first meta-analysis to include only comparative studies investigating the prevalence of MetS in PsA compared to the general population, PsO, and other inflammatory arthropathies. A further strength of this study was that a systematic search was conducted by two librarians. Furthermore, the systematic review was conducted by two independent researchers, with a thorough compilation of the data from the included articles. Results were compiled from several different studies, with differing study populations in terms of inclusion and exclusion criteria, which may provide a better reflection of the actual patient groups and general populations. In this meta-analysis, articles in which the study collected its sample of patients only via a dermatology clinic were excluded, limiting the risk of studies only including patients with very mild arthritic disease.

A weakness of this analysis was that few of the studies comparing the prevalence of MetS in PsA with RA, PsO, and AS were matched in terms of age and sex. The included studies were few in number, and the sample size varied widely between studies. The studies had different outcome measures and different patient population selection strategies, which probably resulted in the populations differing between studies in terms of parameters such as disease activity and treatment. These could be contributing factors that affected especially the heterogeneity, which was high in some of the results. The funnel plot performed on the meta-analyses comparing PsA to PsO showed a somewhat asymmetric picture. This could indicate that there was a publication bias, in which studies with significant results were more likely to be published. There was also insufficient data to conduct relevant meta-analyses on the different components of MetS in the studies comparing PsA with general populations, AS, and PsO.

## Conclusion

This meta-analysis provides further support to the notion that there is an increased prevalence of MetS in PsA compared to the general population and groups with other inflammatory arthropathies. This could be explained by common pathophysiological signaling pathways between psoriatic diseases, obesity, MetS, and endothelial dysfunction, which in turn leads to an increased prevalence of cardiovascular morbidity. It is therefore important to monitor and treat cardiovascular risk factors in these patients, while ensuring that patients achieve minimal disease activity. The impact of disease activity, inflammation, and antirheumatic drugs on the prevalence of MetS in PsA is incompletely understood, and more research is needed in this area on how to best manage the cardiovascular risk in these patients.

## Supplementary Information

Below is the link to the electronic supplementary material.

ESM 1 (DOCX 440 KB)

ESM 2 (DOCX 271 KB)

## Data Availability

The datasets generated during the current study are available from the corresponding author on reasonable request.
