# Supplementary material for: Prevalence of metabolic syndrome and its components in psoriatic arthritis compared with general population, cutaneous psoriasis, and other inflammatory arthropathies: a meta-analysis
Source: Clin Rheumatol. 2025 Aug 18;44(10):3787–99. doi: 10.1007/s10067-025-07637-z (PMC12518441; doi:10.1007/s10067-025-07637-z)
Supplement: Supplementary file 1 — (DOCX 440 KB) [file 10067_2025_7637_MOESM1_ESM.docx]

*Supplement Clinical Rheumatology*

Prevalence of metabolic syndrome and its components in Psoriatic Arthritis compared with general population, cutaneous psoriasis and other inflammatory arthropathies: a meta-analysis.

Sara Andreasson^1^, Anna Södergren^2^

1 Departement of Public Health and Clinical Medicine, Departement of Research and Development- Sundsvall, Umeå University, Umeå, Sweden

2 Department of Public Health and Clinical Medicine, Rheumatology, Umeå University, Sweden

[anna.sodergren@umu.se](mailto:anna.sodergren@umu.se)

**Figure 1:** Search string for Ovid Medline.


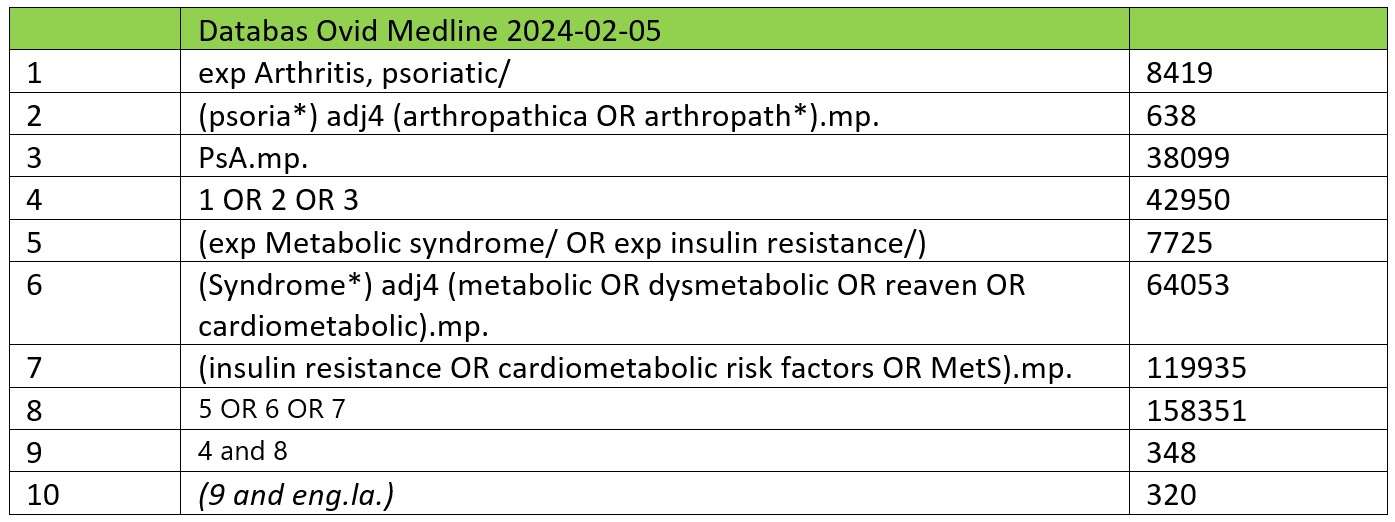


**Figure 2:** Search string for Scopus.


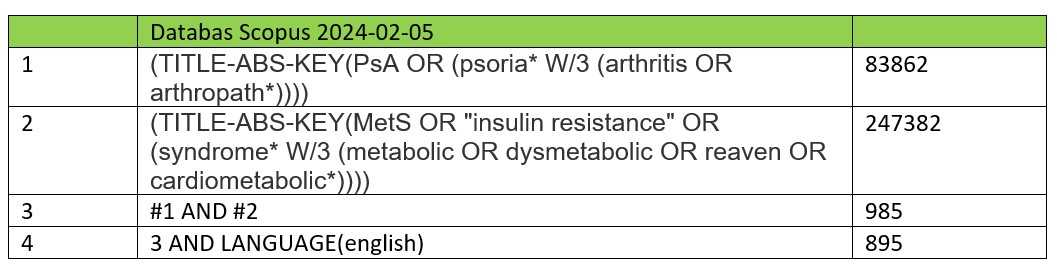


**Figure 3**: Search string for Web of Science.


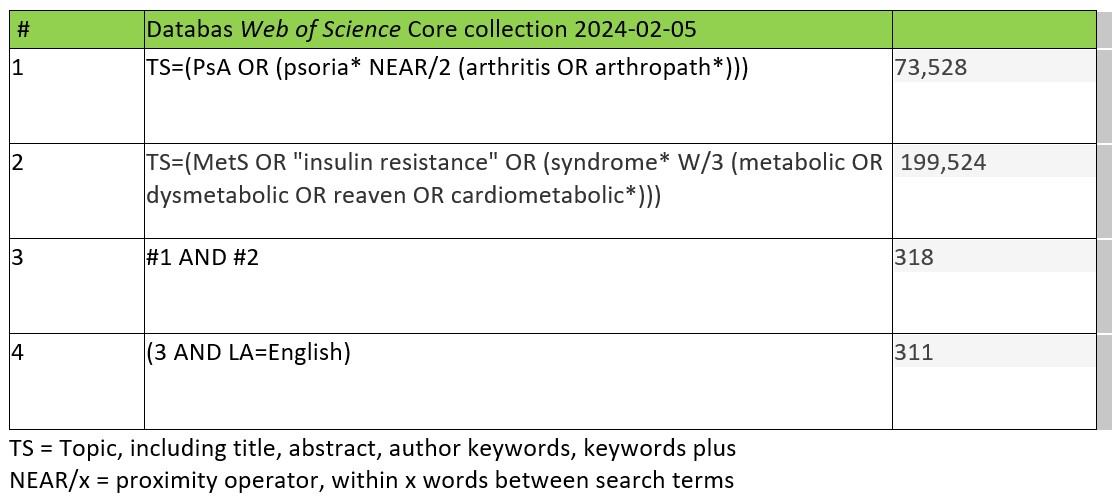


**Table 1:** Overview of the included studies.

| **Study** | **Country** | **Type of study** | **Type of control group** | **Recruitment** | **PsA (n)** | **Control (n)** | **Study size (n)** | **Men (%)** | **Mean age (years)** | **Mean duration of disease PsA (years)** | **Diagnostic criteria PsA** | **Diagnostic criteria MetS** |
| --- | --- | --- | --- | --- | --- | --- | --- | --- | --- | --- | --- | --- |
| **Haroon**  **2016 (1)** | Ireland | Cross-sectional study | General population | Specialist clinic | 283 | 100 | 383 | 47 | 54.6 | 19.4 | CASPAR | AHA/NHLBI |
| **Özkan**  **2017 (2)** | Turkey | Cross-sectional study | RA | Specialist clinic | 102 | 102 | 204 | 29.5 | 44 | 6.5 | CASPAR | NCEP ATP III, IDF |
| **Adiorato Ramos**  **2021 (3)** | Brazil | Cross-sectional study | General population | Specialist clinic | 76 | 76 | 152 | 43.4 | 51 | 6 | CASPAR | Harmonizing |
| **Feld**  **2018 (4)** | Israel | Cross-sectional study | General population | Specialist clinic | 74 | 82 | 156 | 43.2 | 57.6 |  | CASPAR | Harmonizing |
| **Mok**  **2011 (5)** |  | Cross-sectional study | General population, RA, AS | Specialist clinic | 109 | 1065 | 1174 | 50 | 50.4 | 3.6 | CASPAR | Harmonizing |
| **Eder**  **2013 (6)** | Canada | Cross-sectional study | PsC | Registry | 203 | 155 | 358 | 60.6 | 51.6 |  | CASPAR | NCEP ATP III |
| **Labitigan**  **2014 (7)** | USA | Cross-sectional study | RA | Registry | 294 | 1162 | 1456 | 54 | 55.7 | 11.1 | Rheumatologist | Own definition, WHO and ATP III |
| **Tam**  **2008 (8)** | China | Cross-sectional study | General population | Specialist clinic | 102 | 82 | 184 | 47 | 48.7 | 9 | Moll and Wright | NCEP ATP III |
| **Pehlevan**  **2014 (9)** | Turkey | Cross-sectional study | General population | Specialist clinic | 59 | 82 | 141 | 42.4 | 46.8 |  | CASPAR | NCEP ATP III |
| **Atzeni**  **2023 (10)** | Italy | Retrospective observational study | RA | Specialist clinic | 78 | 92 | 170 | 30.8 | 45.23 | 18.86 | CASPAR | AHA/NHLBI |
| **Azevedo**  **2019 (11)** | Portugal | Cross-sectional study | RA | Specialist clinic | 75 | 150 | 225 | 53.3 | 60.7 | 10 | CASPAR |  |
| **Bostoen**  **2014 (12)** | Belgium | Cross-sectional study | PsC | Specialist clinic and a patient organisation | 55 | 49 | 104 | 69.1 | 49.7 | 14.5 | CASPAR | IDF |
| **Cioffi**  **2021 (13)** | Italy | Prospective observational study | RA, AS | Specialist clinic | 134 | 320 | 454 |  |  |  | CASPAR | Harmonizing |
| **Dagdelen**  **2020 (14)** | Turkey | Cross-sectional study | General population, PsC | Specialist clinic | 40 | 140 | 180 | 35 | 49 | 8.2 | CASPAR | AHA/NHLBI |
| **Husni**  **2018 (15)** | USA | Cross-sectional study | PsC | Specialist clinic | 198 | 145 | 343 | 145 | 50.4 | 21.1 | CASPAR | NCEP ATP III |
| **Larid**  **2022 (16)** | France | Cross-sectional study | PsC | Specialist clinic | 37 | 11 | 48 | 43.2 | 51 |  | CASPAR |  |
| **Li 2023 (17)** | China | Cross-sectional study | RA | Specialist clinic | 197 | 279 | 476 | 54.3 | 47.9 | 4 | CASPAR | IDF |
| **Lin**  **2014 (18)** | USA | Cross-sectional study | PsC | Database | 198 | 145 | 343 | 51 | 50.4 | 21.1 | CASPAR | NCEP ATP III |
| **Petcharat**  **2021 (19)** | Thailand | Cross-sectional study | AS | Specialist clinic | 166 | 153 | 319 | 46.4 | 49 | 3 | CASPAR | Harmonizing |
| **Özkul**  **2019 (20)** | Turkey | Cross-sectional study | PsC, RA | Specialist clinic | 55 | 100 | 155 | 29 | 49.9 | 9.5 | CASPAR | IDF |

**Table 2**: List of studies comparing the prevalence of MetS in PsA and general population.

| **Author** | **Definition of control group** | **MetS diagnostic criteria** | **Matching** | **PsA (n)** | **Control group (n)** | **PsA MetS (%)** | **Control MetS (%)** | **P-value** |
| --- | --- | --- | --- | --- | --- | --- | --- | --- |
| **Haroon**  **2016 (1)** | Patients with non-inflammatory rheumatic disease. | AHA/NHLBI | Age and sex. | 283 | 100 | 44 | 29 | 0.0009 |
| **Adiorato Ramos**  **2021 (3)** | Without PsA or connective tissue disease. | Harmonizing (IDF/NHLBI/AHA/WHF/IAS/IASO) | Age and sex. | 76 | 76 | 53.9 | 18.4 | 0.001 |
| **Feld**  **2018 (4)** | Without psoriasis, inflammatory arthritis or other supporting tissue diseases recruited from primary healthcare centers. | Harmonizing (IDF/NHLBI/AHA/WHF/IAS/IASO) | Age and sex. | 74 | 82 | 54.8 | 36.6 | 0.02 |
| **Mok**  **2011 (5)** | No specific definition. | Harmonizing (IDF/NHLBI/AHA/WHF/IAS/IASO) | Age and sex. | 109 | 244 | 38 | 17 | 0.001 |
| **Tam**  **2008 (8)** | Healthy controls were recruited from hospital staff. | NCEP ATP III | Age, sex, and ethnicity. | 102 | 82 | 23.5 | 15.9 | 0.19 |
| **Pehlevan**  **2014 (9)** | Healthy volunteers. | NCEP ATP III | No matching. Control group was younger. No significant different in terms of sex. | 59 | 82 | 35.5 | 14.6 | 0.004 |
| **Dagdelen**  **2020 (14)** | Patients seeking care for cosmetic reasons. | AHA/NHLBI | Age and sex. | 40 | 60 | 48 | 28 | 0.028 |

**Table 3**: List of studies comparing the prevalence of MetS in PsA and RA.

| **Author** | **Diagnosis criteria RA** | **Diagnostic criteria MetS** | **Matching** | **PsA (n)** | **RA (n)** | **PsA MetS (%)** | **RA MetS (%)** | **P-value** |
| --- | --- | --- | --- | --- | --- | --- | --- | --- |
| **Mok**  **2011 (5)** | ACR | Harmonizing | No matching.  Similar age. More women in the RA group. | 109 | 699 | 38 | 20 | <0.001 |
| **Özkan**  **2017 (2)** | EULAR | NCEP ATP III, IDF | Age and sex. | 102 | 102 | 46.8 | 27.9 | 0.0019 |
| **Labitigan**  **2014 (7)** | Rheumatologist | Own definition, WHO and ATP III | Adjusted for age, sex, and ethnicity. | 294 | 1162 | 27 | 19 | 0.02 |
| **Atzeni**  **2023 (10)** | ACR/EULAR | AHA/NHLBI | No matching.  Significant difference in age, but not in sex. | 78 | 92 | 51.3 | 27.2 | 0.002 |
| **Azevedo**  **2019 (11)** | ACR/EULAR |  | No matching.  No significant different in terms of age. Significantly more women in the RA group. | 75 | 150 | 56 | 32 | 0.059 |
| **Cioffi**  **2021 (13)** | ACR/EULAR | Harmonizing | Not reported. | 134 | 228 | 19 | 15 | >0.1 |
| **Li**  **2023 (17)** | ACR/EULAR | IDF | Age and sex. | 197 | 279 | 36 | 23.3 | 0.002 |
| **Özkul**  **2019 (20)** | ACR/EULAR | IDF | No matching.  Significant difference in age and sex. The RA group was older and had a larger propotion of women. | 55 | 50 | 29.1 | 36 | 0.684 |

**Table 4:** List of studies comparing the prevalence of MetS in PsA and AS.

| **Author** | **Diagnosis criteria AS** | **Diagnostic criteria MetS** | **Matching** | **PsA (n)** | **AS (n)** | **MetS PsA (%)** | **MetS AS (%)** | **P-value** |
| --- | --- | --- | --- | --- | --- | --- | --- | --- |
| **Mok**  **2011 (5)** | Modified New York criteria | Harmonizing | No matching. The AS group was younger and had a larger proportion of men. | 109 | 122 | 38 | 11 | **<0.001** |
| **Cioffi**  **2021 (13)** | ASAS | Harmonizing | Not reported. | 134 | 96 | 19 | 15 | >0.1 |
| **Petcharat**  **2021 (19)** | Modified New York criteria | Harmonizing | No matching. The AS group was younger and had a larger proportion of men. | 166 | 153 | 43.4 | 19 | <0.001 |

**Table 5:** List of studies comparing the prevalence of MetS in PsA and PsC

| **Study** | **Diagnosis criteria PsC** | **Diagnostic criteria MetS** | **Matching** | **PsA (n)** | **PsC (n)** | **PsA MetS (%)** | **PsC MetS (%)** | **P-value** |
| --- | --- | --- | --- | --- | --- | --- | --- | --- |
| **Eder**  **2013 (6)** | Diagnosed by a dermatologist | NCEP ATP III | Age and sex. | 203 | 155 | 36.5 | 27.1 | 0.056 |
| **Bostoen**  **2014 (12)** | Diagnosed by a dermatologist | IDF | No matching. No significant difference in age. The PsA group had a larger proportion of men. | 55 | 49 | 25.5 | 44.9 | 0.039 |
| **Dagdelen**  **2020 (14)** | Not reported | AHA/NHLBI | No matching. The PsA group was older and had a smaller proportion of men. | 40 | 80 | 48 | 49 | >0.05 |
| **Husni**  **2018 (15)** | Diagnosed by a dermatologist | NCEP ATP III | No matching. The PsA group was slightly older. No significant different in terms of sex. | 198 | 145 | 42 | 28.9 | 0.024 |
| **Larid**  **2022 (16)** | Diagnosed by a dermatologist |  | Age and sex. | 37 | 11 | 32.3 | 36.4 | >0.999 |
| **Lin**  **2014 (18)** | Diagnosed by a dermatologist | NCEP ATP III | No matching. The PsA group was slightly older. No significant different in terms of sex. | 198 | 145 | 42 | 28.9 | 0.024 |
| **Özkul**  **2019 (20)** | Diagnosed by a dermatologist | IDF | No matching. The PsA group was slightly older and had a larger proportion of women. | 55 | 50 | 29.1 | 36 | 0.684 |

1. Haroon M, Rafiq Chaudhry AB, Fitzgerald O. Higher Prevalence of Metabolic Syndrome in Patients with Psoriatic Arthritis: A Comparison with a Control Group of Noninflammatory Rheumatologic Conditions. Journal of Rheumatology. 2016;43(2):463-4.

2. Özkan SG, Yazisiz H, Behlül A, Gökbelen YA, Borlu F, Yazisiz V. Prevalence of metabolic syndrome and degree of cardiovascular disease risk in patients with Psoriatic Arthritis. EUROPEAN JOURNAL OF RHEUMATOLOGY. 2017;4(1):40-5.

3. Adeodato Ramos LM, Gomes KWP, De Saboia Mont'alverne AR, Braga MV, Costa Vasconcelos AH, Rodrigues CEM. High Prevalence of Metabolic Syndrome in Patients With Psoriatic Arthritis From Northeastern Brazil: Association With Traditional Cardiovascular Risk Factors and Biologic Disease-Modifying Antirheumatic Drugs. Journal of Clinical Rheumatology. 2021;27:S186-S92.

4. Feld J, Nissan S, Eder L, Rahat MA, Elias M, Rimar D, et al. Increased Prevalence of Metabolic Syndrome and Adipocytokine Levels in a Psoriatic Arthritis Cohort. JCR: Journal of Clinical Rheumatology. 2018;24(6):302-7.

5. Mok CC, Ko GT, Ho LY, Yu KL, Chan PT, To CH. Prevalence of atherosclerotic risk factors and the metabolic syndrome in patients with chronic inflammatory arthritis. Arthritis care & research. 2011;63(2):195-202.

6. Eder L, Jayakar J, Pollock R, Pellett F, Thavaneswaran A, Chandran V, et al. Serum adipokines in patients with psoriatic arthritis and psoriasis alone and their correlation with disease activity. Annals of the Rheumatic Diseases. 2013;72(12):1956-61.

7. Labitigan M, Bahce-Altuntas A, Kremer JM, Reed G, Greenberg JD, Jordan N, et al. Higher rates and clustering of abgeneral lipids, obesity, and diabetes mellitus in psoriatic arthritis compared with rheumatoid arthritis. Arthritis care & research. 2014;66(4):600-7.

8. Tam LS, Tomlinson B, Chu TTW, Li M, Leung YY, Kwok LW, et al. Cardiovascular risk profile of patients with psoriatic arthritis compared to controls - The role of inflammation. Rheumatology. 2008;47(5):718-23.

9. Pehlevan S, Yetkin DO, Bahadir C, Goktay F, Pehlevan Y, Kayatas K, Ince N. Increased prevalence of metabolic syndrome in patients with psoriatic arthritis. Metabolic Syndrome & Related Disorders. 2014;12(1):43-8.

10. Atzeni F, La Corte L, Cirillo M, Giallanza M, Galloway J, Rodríguez-Carrio J. Metabolic Syndrome and Its Components Have a Different Presentation and Impact as Cardiovascular Risk Factors in Psoriatic and Rheumatoid Arthritis. Journal of Clinical Medicine. 2023;12(15).

11. Azevedo S, Santos-Faria D, Leite Silva J, Ramos Rodrigues J, Sousa Neves J, Peixoto D, et al. Obesity, metabolic syndrome and other comorbidities in rheumatoid arthritis and psoriatic arthritis: influence on disease activity and quality of life. Acta Reumatologica Portuguesa. 2019;44(4):322-4.

12. Bostoen J, Van Praet L, Brochez L, Mielants H, Lambert J. A cross-sectional study on the prevalence of metabolic syndrome in psoriasis compared to psoriatic arthritis. Journal of the European Academy of Dermatology & Venereology. 2014;28(4):507-11.

13. Cioffi G, Viapiana O, Tarantini L, Orsolini G, Idolazzi L, Sonographer FO, et al. Clinical profile and outcome of patients with chronic inflammatory arthritis and metabolic syndrome. Internal & Emergency Medicine. 2021;16(4):863-74.

14. Dagdelen D, Karadag AS, Kasapoglu E, Wang JV, Erman H. Correlation of metabolic syndrome with serum omentin-1 and visfatin levels and disease severity in psoriasis and psoriatic arthritis. Dermatologic Therapy. 2020;33(6):e14378.

15. Husni ME, Wilson Tang WH, Lucke M, Chandrasekharan UM, Brennan DM, Hazen SL. Correlation of High-Density Lipoprotein-Associated Paraoxonase 1 Activity With Systemic Inflammation, Disease Activity, and Cardiovascular Risk Factors in Psoriatic Disease. Arthritis & Rheumatology. 2018;70(8):1240-50.

16. Larid G, Delwail A, Dalle T, Vasseur P, Silvain C, Jegou JF, et al. Ex vivo cytokine production in psoriatic disease: Towards specific signatures in cutaneous psoriasis and peripheral psoriatic arthritis. Frontiers in Immunology. 2022;13:993363.

17. Li B, Huang H, Zhao J, Deng X, Zhang Z. Discrepancy in Metabolic Syndrome between Psoriatic Arthritis and Rheumatoid Arthritis: a Direct Comparison of Two Cohorts in One Center. Rheumatology and Therapy. 2023;10(1):135-48.

18. Lin YC, Dalal D, Churton S, Brennan DM, Korman NJ, Kim ES, Husni ME. Relationship between metabolic syndrome and carotid intima-media thickness: cross-sectional comparison between psoriasis and psoriatic arthritis. Arthritis care & research. 2014;66(1):97-103.

19. Petcharat C, Srinonprasert V, Chiowchanwisawakit P. Association between syndesmophyte and metabolic syndrome in patients with psoriatic arthritis or ankylosing spondylitis: a cross-sectional study. BMC Musculoskeletal Disorders. 2021;22(1):367.

20. Özkul Ö, Yazici A, Aktürk SS, Karadag DT, Isik OO, Tekeoglu S, Cefle A. Are there any differences among psoriasis, psoriatic arthritis and rheumatoid arthritis in terms of metabolic syndrome and cardiovascular risk factors? EUROPEAN JOURNAL OF RHEUMATOLOGY. 2019;6(4):174-8.
